# Characterization of an Emerging Recombinant Duck Circovirus in Northern Vietnam, 2023–2024

**DOI:** 10.3390/v17050732

**Published:** 2025-05-20

**Authors:** Hieu Van Dong, Dai Quang Trinh, Giang Huong Thi Tran, Thanh Thi Vu, Thinh Hung Ba Nguyen, Amonpun Rattanasrisomporn, Dao Anh Tran Bui, Jatuporn Rattanasrisomporn

**Affiliations:** 1 Faculty of Veterinary Medicine, Vietnam National University of Agriculture, Trau Quy Town, Gia Lam District, Hanoi 131000, Vietnam; dvhieuvet@vnua.edu.vn (H.V.D.); giangtranvet@vnua.edu.vn (G.H.T.T.); vtthanh@gmail.com (T.T.V.); hungthinhnguyenba@gmail.com (T.H.B.N.); btadao@vnua.edu.vn (D.A.T.B.); 2 Central Veterinary Medicine JSC No. 5, Ha Binh Phuong Industrial Zone, Hanoi 131000, Vietnam; trinhquangdai82@gmail.com; 3 Interdisciplinary of Genetic Engineering and Bioinformatics, Graduate School, Kasetsart University, Bangkok 10900, Thailand; fgraapr@ku.ac.th; 4 Department of Companion Animal Clinical Sciences, Faculty of Veterinary Medicine, Kasetsart University, Bangkok 10900, Thailand

**Keywords:** complete genome, circovirus, ducks, Vietnam

## Abstract

This study aimed to characterize the duck circovirus circulating in Northern Vietnam based on complete genome sequences. Between 2023 and 2025, 45 pooled tissue samples were collected from nine duck flocks in several provinces in Northern Vietnam. Of the 45 samples tested, 16 (35.56%) were positive for the DuCV genome, as determined using conventional polymerase chain reaction. Nine representative strains were selected for viral genome sequencing. The results indicated that the complete Vietnamese DuCV genomes were from 1992 to 1995 bp in length, and the degree of nucleotide identity shared among them ranged from 96.88% to 99.84%. Phylogenetic analysis of the complete genomes showed that the nine Vietnamese DuCV strains belonged to genotype I, subgenotypes Ia (two strains), Ib (four strains), and Ic (three strains). These viral strains were genetically related to viruses reported in China from 2019 to 2023. Recombination events occurred on the Cap gene sequences of three Vietnamese DuCV strains (Vietnam/VNUA-102/2023, Vietnam/VNUA-225/2023, and Vietnam/VNUA-318/2024). One positive selection was detected on the Rep protein sequence.

## 1. Introduction

Duck circovirus (DuCV) belongs to the Circoviridae family and includes two genera, Cyclovirus and Circovirus, according to a report by the International Committee on Taxonomy of Viruses (ICTV) released in 2022. The Circovirus genus consists of porcine circovirus types 1–4, canine circovirus, and some strains in bats. Regarding avian hosts, the Circovirus genus includes psittacine beak and feather disease virus, goose circovirus, and DuCV. DuCV is a small, non-enveloped virus that contains single-stranded circular DNA [1,2].

DuCV is a contagious immunosuppressive virus; it harms many duck species and has caused several outbreaks in the poultry industry worldwide. DuCV-infected ducks show stunted growth, abnormal feathering, weakened immune systems, and a susceptibility to other diseases, such as *Riemerella anatipestifer*, avian pathogenic *Escherichia coli*, *Pastereulla multocida*, and *Salmonella*. Therefore, DuCV poses a significant economic challenge to the duck industry [3,4].

DuCV is described as having icosahedral symmetry and ranges from approximately 15 to 16 nm in diameter. It is considered to be the smallest duck virus. The DuCV genome ranges from 1988 to 1996 nucleotides (nt) in length [5,6]. It contains three open reading frames (ORFs) and two intergenic noncoding regions [2]. In detail, ORF1 encodes the Rep protein, which is needed for viral replication. It is stated that the Rep protein sequence is highly conserved among various DuCV genotypes. ORF2 encodes the Cap protein, which is the viral structural and virulence-associated protein involved in the host immune response. The remaining viral non-structural protein is encoded by the DuCV ORF3 gene. The protein is regarded as the source of DuCV’s apoptotic activity, depending on the cell line [2,7,8].

DuCV is divided into two main genotypes, DuCV-1 and DuCV-2, depending on the phylogenetic tree of the full-length genome and the cap gene [9,10]. In brief, the DuCV-1 genotype is classified into DuCV-1a and DuCV-1b, while the DuCV-2 genotype contains DuCV-2a, DuCV-2b, and DuCV-2c. Furthermore, DuCV-3, as a new type, was reported in laying ducks in Hunan Province in China in 2022. The DuCV-3 genome is about 1755 nt in length. Its shared nt identity with other DuCV genotypes ranges from 62.3% to 63.7% [5].

DuCV was first reported in a six-week-old duck flock in Germany in 2003. Clinical symptoms, including disheveled plumage, emaciation, immunosuppression, and weight loss, were observed in the diseased ducks. Afterward, DuCV surveillance was carried out in many countries in the world, such as Hungary, Taiwan (China), the United States, China, Thailand, etc. [6,8,11,12,13,14,15,16]. It is worth noting that DuCV has been reported to be highly infectious among Chinese breeder flocks and leads to viral persistence in these flocks [17,18].

In Vietnam, DuCV was first reported in duck flocks raised in northern areas in 2022 [19]. DuCV strains, including DuCV-1 and DuCV-2, were circulating in commercial flocks. However, there are few reports investigating the evolution of DuCV in Vietnam. In addition, continuous surveillance of the DuCV genotype prevalence in duck flocks is necessary to better understand viral genetic diversity. Therefore, this study aimed to deepen our understanding of the evolutionary dynamics of DuCV circulating in commercial duck flocks in Vietnam.

## 2. Materials and Methods

### 2.1. Ethics Statement

The Committee on Animal Research and Ethics of the Vietnam National University of Agriculture (CARE-2023/08) approved this study. All protocols involving animals in this study were used after obtaining ethics approval from the Committee on Animal Research and Ethics of the Vietnam National University of Agriculture. Informed consent was acquired from the duck owners before sampling and data publication.

### 2.2. Samples

In this study, a total of 9 duck farms in several provinces in Northern Vietnam, including Thainguyen, Bacgiang, Haiduong, Nghean, Phutho, Hanoi, and Hanam, were selected for sampling during 2023 and 2024. Samples were collected from ducks aged 2 to 7 weeks across nine farms, with five ducks sampled from each farm. The selected farms showed signs of increased mortality rates and decreased growth. Selected ducks from each farm were sent to the Faculty of Veterinary Medicine, Vietnam National University of Agriculture. Pooled tissue samples, including the liver, heart, brain, and bursa of Fabricius, were collected and then homogenized in phosphate-buffered saline and stored at −80 °C until use.

### 2.3. DNA Extraction and Polymerase Chain Reaction (PCR)

DNA was extracted from tissue homogenates using a Viral Gene-spin^TM^ Viral DNA/RNA Extraction Kit (iNtRON Biotechnology, Seoul, Republic of Korea) according to the manufacturer’s directions. The DNA was eluted in 50 µL of distilled water.

For detection, a DuCV-F1/R1 primer set (DuCV-F1, CCC GCC GAA AAC AAG TAT TA-3′; DuCV-R1, 5′-TCG CTC TTG TAC CAA TCA CG-3′) was used to amplify the target 230 bp part of the DuCV Rep gene as described previously [15]. The following thermal conditions were used: an initial denaturation step at 94 °C for 5 min, followed by 35 cycles at 94 °C for 45 s, 45 °C for 30 s, 72 °C for 30 s, and a final extension step at 72 °C for 10 min. PCR products with 230 bp were examined in a 1.2% agarose gel and then detected by UV light [15].

Regarding complete genome sequencing, four primer sets were used to amplify the 887 bp, 997 bp, 492 bp, and 802 bp fragments of the DuCV genome [19,20]. The following thermal conditions were used: an initial denaturation step at 95 °C for 5 min, followed by 34 cycles at 95 °C for 30 s, 55 °C (four pairs of primers) for 30 s, and 72 °C for 40 s and a final extension step at 72 °C for 10 min. The PCR products were examined in a 1.2% agarose gel and then detected by UV light [19,20].

### 2.4. Nucleotide Sequencing and Analysis

PCR products were purified using GeneClean^®^ II Kits (MP Biomedicals, Santa Ana, CA, USA). Sequencing of the Vietnamese DuCV genomes was performed by 1st BASE, Selangor, Malaysia.

The sequence data were analyzed by GENETYX ver. 10 software (GENETYX Corp., Tokyo, Japan). The multiple DuCV sequences available (Table 1) were compared with the DuCV sequences in this study using BLAST version 1.3 homology searches. The Clustal W algorithm in BioEdit version 7.2 was used to perform the nucleotide identity comparisons of the complete genomes and full-length Rep and Cap genes and to deduce the amino acid substitution of the Rep and Cap proteins [21,22]. Phylogenetic trees were created using the Maximum Likelihood method supported by 1000 bootstrap replicates in MEGA X software “https://www.megasoftware.net/” (accessed on 20 January 2025). The sequence data obtained in this study were deposited into GenBank with accession numbers PV483400 to PV483408.

### 2.5. Recombination Events and Selection Profiles of Vietnamese DuCV Strains

Putative recombinations were identified using a recombination detection program (RDP) version Beta 4.97 [23] and SimPlot software version 3.5.1 [24]. The elaboration of evolutionary selection profiles was performed using Datamonkey “http://www.datamonkey.org/” (accessed on 28 January 2025) by following the Fast Unconstrained Bayesian AppRoximation (FUBAR) method [25]. The general time-reversible model was selected based on the best-fit evolutionary model test.

## 3. Results

### 3.1. Detection of Duck Circovirus in Field Samples

The results indicated that 16 (35.56%) out of 45 samples were positive for the viral genome. All farms tested were positive for DuCV infection. The proportion of positive cases observed across the tested locations/farms ranged from 20% to 60% (Table 2).

### 3.2. Characterization of Vietnamese DuCV Genomes

A total of nine representative DuCV-positive samples were selected for viral genome sequencing. Complete genome sequences of DuCV strains were successfully sequenced and characterized. The nine strains were designated as Vietnam/VNUA-102/2023, -114/2023, -137/2023, -225/2023, -251/2023, -318/2024, -322/2024, -315/2024, and -331/2024. The length of the Vietnamese complete genomes ranged from 1992 to 1995 nucleotides. Neither deletion nor insertion mutations were found in the coding regions of the viral genomes in this study.

The nucleotide identities of the complete genomes among the Vietnamese DuCV strains obtained in this study and previous Vietnamese strains are indicated in Table 3 and Table 4. Among current DuCV strains, nucleotide identity ranged from 96.88% to 99.84% (Vietnam/VNUA-137/2023 vs. Vietnam/VNUA-315/2024) (Table 3). The nucleotide identity between the strains in this study and previously reported strains ranged from 83.40% (Vietnam/VNUA-315/2024 vs. Vietnam/OM176556.1/VNUA-HD89/2021) to 99.79% (Vietnam/VNUA-318/2024 vs. Vietnam/OM176555.1/VNUA-TN85/2021) (Table 4).

The nine Vietnamese DuCV strains in this study were compared with strains from other countries found in GenBank. The Vietnamese DuCV genome sequences shared the highest nucleotide identities with those of Chinese strains (DY01, GX05, GX11, GX25, GX39, GX51, and CN/GD/1092), ranging from 99.24% to 99.74% (Table 5).

**Figure 1:**
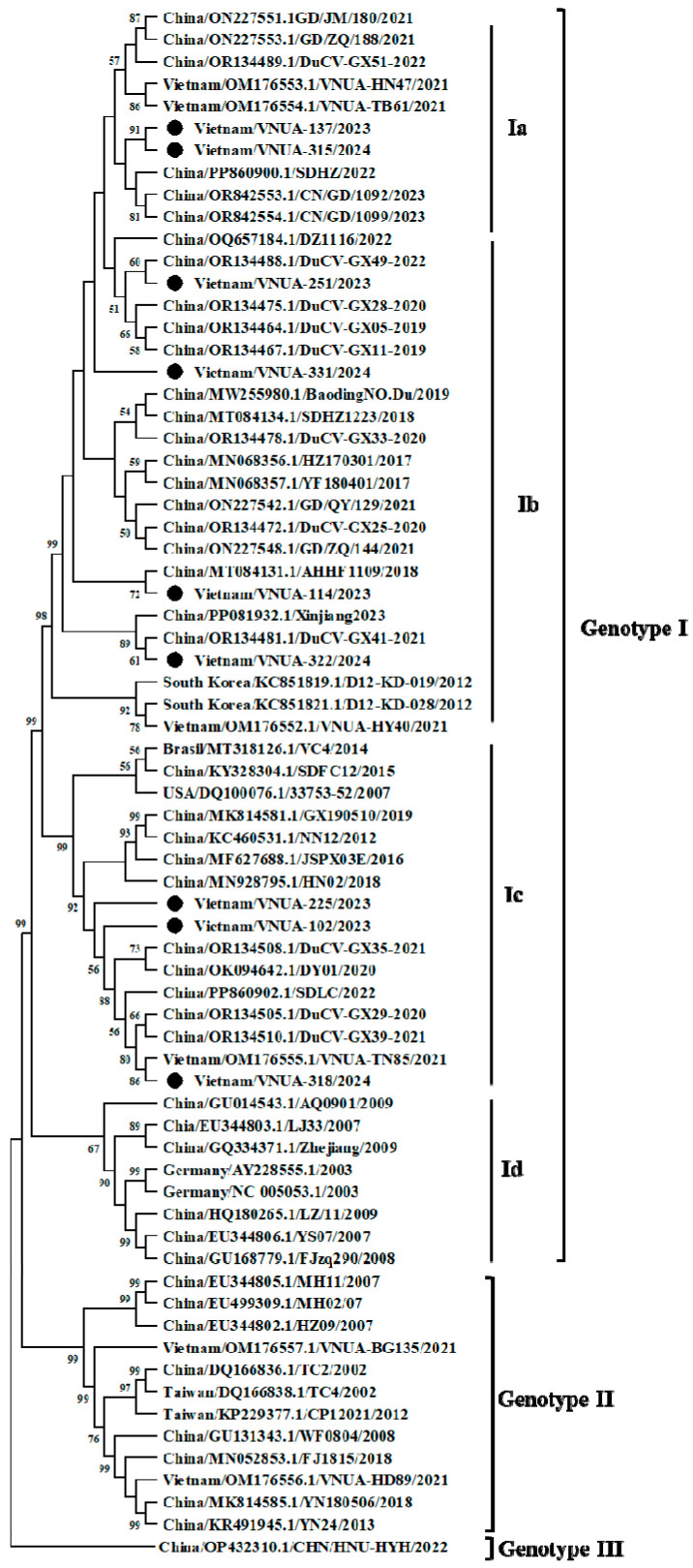
A phylogenetic tree was constructed based on the complete genome sequences of Vietnamese DuCV strains compared with the strains available from GenBank. The maximum likelihood method with the Kimura-2 parameter model was used in MEGA X software to establish the phylogenetic tree (1000 bootstrap replicates). The Vietnamese DuCV strains in this study are marked by solid circles.

Phylogenetic analysis indicated that the nine DuCV strains obtained belonged to genotype I. Among the nine strains, several subgenotype viruses were found, including subgenotype Ia (2 strains), Ib (4 strains), and Ic (3 strains) (Figure 1).

**Figure 2:**
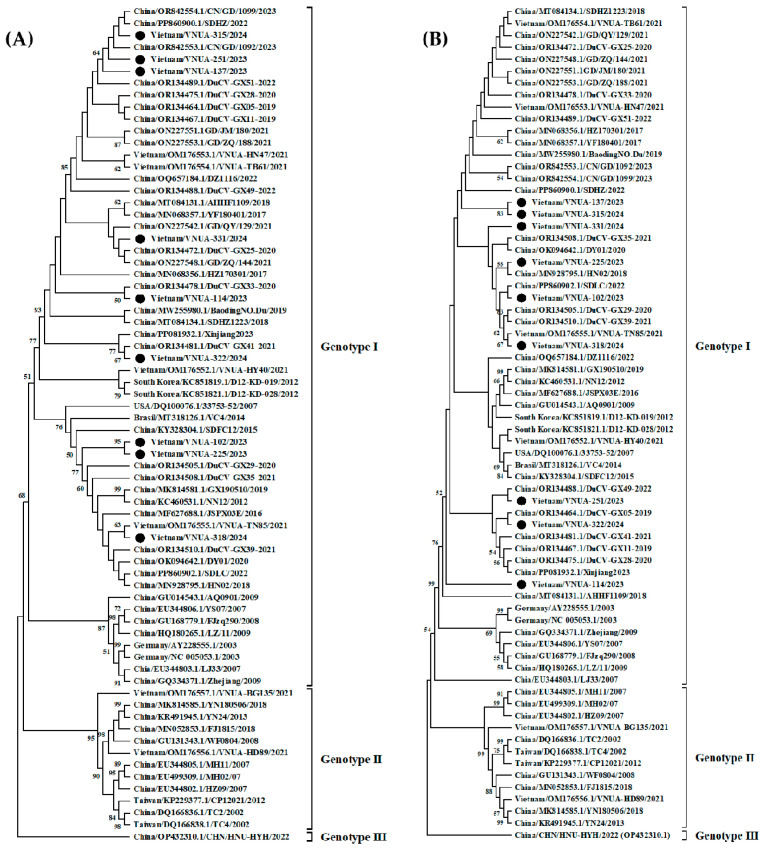
Phylogenetic trees were established based on the full-length (**A**) Cap sequences and (**B**) Rep sequences of Vietnamese DuCV strains compared with the strains available from GenBank. The maximum likelihood method with the Kimura-2 parameter model was used in MEGA X software to establish the phylogenetic trees (1000 bootstrap replicates). The Vietnamese DuCV strains in this study are marked by solid circles.

Analysis of Cap gene and protein sequences: Among the Vietnamese DuCV strains obtained in this study, nucleotide identity ranged from 93.28% (VNUA-102/2023 vs. VNUA-137/2023) to 99.61% (VNUA-225/2023 vs. VNUA-102/202) (Supplementary Table S1). A phylogenetic tree (Figure 2A) was established based on the full-length Cap gene (780 bp) and indicated that the nine Vietnamese DuCV strains in this study clustered into Genotype I.

The deduced amino acids of the Cap protein were also predicted and analyzed. The results indicated that 14 amino acid substitutions were found in the Vietnamese DuCV strains (Table 6). The motif of the amino acid substitutions was 12G/S/A-47N/H-55S/N-82Q/R-106N/S/G-107K/T-155T/S-156T/A-177V/I-183V/I-194T/G-197Y/H-205R/K-236D/N/E. The substitution at residue 156 (T to A) was unique in that it was only detected in two Vietnamese DuCV strains (VNUA-102/2023 and VNUA-225/2023).

Analysis of Rep gene and protein sequences: Among the Vietnamese DuCV strains in this study, nucleotide identity ranged from 99.08% (Vietnam/VNUA-114/2023 vs. Vietnam/VNUA-102/2023) to 100% (Vietnam/VNUA-137/2023 vs. Vietnam/VNUA-315/2024). Analysis of the phylogenetic tree based on the full-length Rep gene sequences showed that the nine Vietnamese DuCV strains in this study belonged to Genotype I.

### 3.3. Recombination Analysis of Vietnamese DuCV Strains

**Figure 3:**
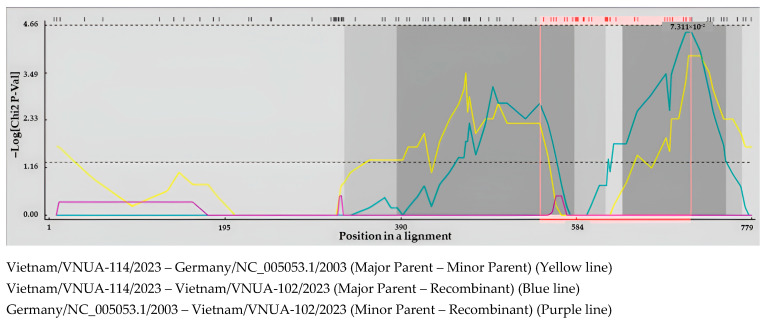
Detection of recombination events of full-length Cap gene sequences of the Vietnam/VNUA-114/2023 DuCV strain performed using MaxChi analysis. A pairwise distance model with window size 200, step size 20, and 1000 bootstrap replicates was generated by the RDP 5 program.

Recombination analysis was performed using the nine Vietnamese DuCV strains in this study, six previous Vietnamese strains, and other strains from GenBank. The results indicated that three recombination events had occurred in the Cap gene sequences of the Vietnamese DuCV strains circulating in Northern Vietnam, whereas no recombination events were found in the remaining gene sequences. The three recombination events were Vietnam/VNUA-102/2023, Vietnam/VNUA-225/2023, and Vietnam/VNUA-318/2024, as determined with the five methods supplemented in RDP 5 software (*p*-value < 0.05) (Table 7 and Table 8). The major parents were the Vietnamese DuCV strains circulating in Vietnam in 2021 (Vietnam/OM176552.1/VNUA-HY40/2021) and 2023 (Vietnam/VNUA-114/2023) (Figure 3), while the minor parent was Germany/NC_005053.1/2003.

**Figure 4:**
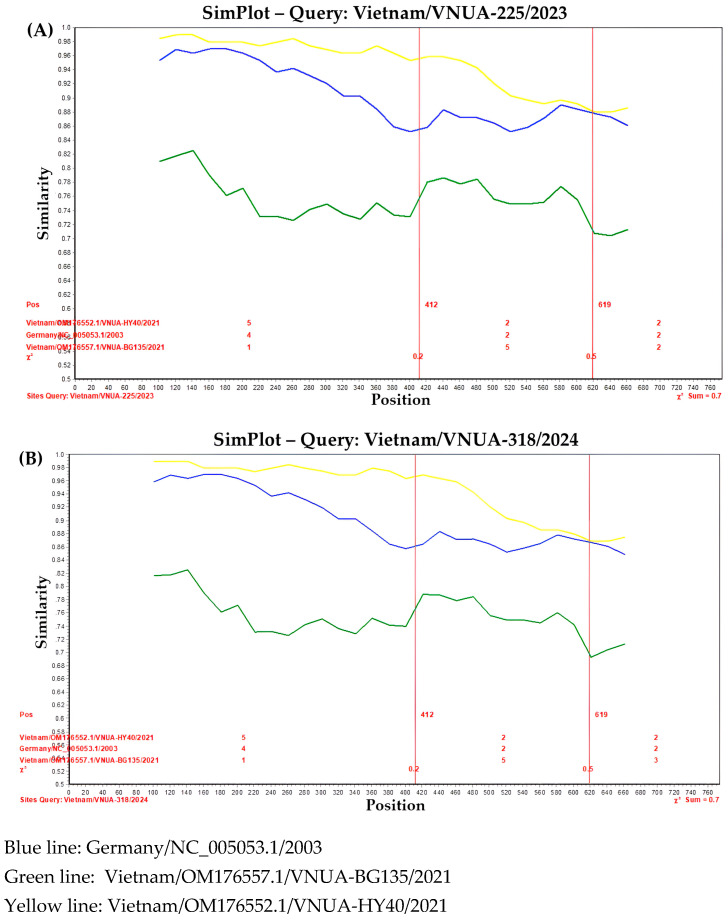
A distance plot was used for recombination identification. The (**A**) Vietnam/VNUA-225/2023 and (**B**) Vietnam/VNUA318/2024 strains were used as a query. Pairwise distance with a window size of 200 bp and step size of 20 bp was applied via the SimPlot program.

To confirm the recombination events, the Vietnam/VNUA-225/2023 or Vietnam/VNUA318/2024 strains were used as a query. Simplot software confirmed that these two strains were recombination events. The breakpoint was at site 619 when the chi-square value changed from 0.2 to 0.5 (Figure 4).

### 3.4. Analysis of Natural Selection Profile of DuCV Gene Sequences

In a previous study, we reported the detection of positive selection at site 106 in the Cap protein. In this study, the same result was found in the Cap protein sequences of the Vietnamese DuCV strains. For Rep protein sequences, evolutionary analysis indicated that one positive selection was found at residue 112 (Table 9), while 87 negative selections were identified (Supplementary Table S3).

## 4. Discussion

In this study, DuCV genomes were detected in 16 (35.56%) samples collected from suspected ducks in Northern Vietnam. Nine DuCV complete genomes were characterized. The nine Vietnamese DuCV strains shared a high degree of nucleotide identity (96.88% to 99.84%), belonged to genotype I, and clustered with Chinese strains. In the present study, we reported recombination events in Vietnamese DuCV strains for the first time. The recombination events among Vietnamese DuCV strains circulating in Northern Vietnam from 2023 to 2024 were seen in the Cap gene sequence, and one positive selection was seen on the Rep protein sequence.

In our previous report on DuCV infection in Northern Vietnam, it was pointed out that the DuCV-positive rate found among ducks in 2021 was high [19]. In this study, we continued to observe DuCV infection among duck flocks in some provinces in Northern Vietnam. In the present study, 35.56% of the tested ducks were positive for the DuCV genome, a number lower than the 43.08% reported in the country in 2021 and the 84% reported in Hungary [15] but higher than the 21.8% observed in the Republic of Korea and the 6.06% seen in the U.S. [8,11]. The low sample size, time constraints, and regional sampling are the main reasons for these differences. The results of this study indicate that DuCV infection remains prevalent in Northern Vietnam, with a detection rate of 35.56%.

Historically, DuCV strains were mainly divided into genotype I (subgenotypes Ia, Ib, Ic, and Id) and genotype II based on the full-length Cap gene sequences and complete genome sequences [5,6,19,26]. A later report confirmed a novel DuCV genotype III virus in mainland China [5]. This finding suggests that DuCV strains worldwide are continuously evolving. Therefore, studies on DuCV infection and genetic characterization are necessary. In this study, we found that DuCV strains circulating in Northern Vietnam belong to genotype I, subgenotypes Ia, Ib, and Ic, which is similar to our previous report from 2021 [19]. Other genotype II and III viruses were not detected in the present study, suggesting that genotype I viruses are the predominant ones in circulation in Northern Vietnam, affecting the duck production industry. The nine Vietnamese DuCV strains in this study and six previously reported strains [19] were genetically close to Chinese DuCV strains. Vietnam and China share a long border, along which live birds, including ducks, are traded daily. The trading of live birds may result in outbreaks of avian influenza in poultry [27,28]. This situation may affect the diversity of DuCV strains in Northern Vietnam.

Recombination is an important pattern of viral evolution. Previously, many researchers reported on recombination events among global DuCV strains, showing that recombination events may occur in both coding and noncoding regions of the viral genome [6,10,11,29]. In our 2021 report on six Vietnamese DuCV strains, we did not find any recombination events. In the present study, three Vietnamese DuCV strains were found to have resulted from recombination events in the Cap gene sequences. Interestingly, the major parents were Vietnamese strains circulating in Northern Vietnam in 2021 and 2023. This finding suggests that recombination plays a critical role in viral evolution, leading to a significant increase in the genetic diversity of DuCV strains.

It was reported that amino acid substitutions occurring in the Cap protein at residues 3–15, 104–124, and 232–238 may have resulted in antigenic changes and viral virulence. In this study, we only detected one positive selection at residue 106, which was similar to our previous report [19]. Rep and ORF3 gene sequences play critical roles in the replication and apoptosis of DuCV strains in host cells [2,7]. Substitutions on the ORF3 protein may result in changes in pathogenesis and immunosuppression [7]. In this study, one positive selection was found at residue 112 on the Rep protein, equivalent to residue 7 on the ORF3 protein, since the ORF3 gene sequence is within the Rep gene sequence, which belongs to one of the immunogenic regions (3–15, 104–124, 232–238) [30]. This finding suggests that virulence gene sequences are under positive selection, in line with a previous report [31]. In addition, 87 negatively selected sites were found among Vietnamese DuCV strains, suggesting that this protein is under purifying selection. In other viruses, virulence genes seem to be under positive selection compared to other genes. Wang et al. pointed out that the Rep gene was more conserved due to strong purifying [30]. This may be due to its critical role in viral replication. Further studies should focus on elucidating the role of natural selection in the Rep and ORF3 genes of DuCV strains.

## 5. Conclusions

In this study, DuCV infection was continuously detected among duck flocks in Northern Vietnam during 2023–2024, with a positive rate of 35.56%. Nine representative complete genomes, ranging from 1992 to 1995 bp in length, were successfully sequenced and characterized. Neither insertions nor deletions were found in the coding regions of these viral genomes. The Vietnamese DuCV complete genomes in this study shared a high degree of nucleotide identity, ranging from 96.88% to 99.84%. Phylogenetic analysis indicated that the nine Vietnamese DuCV strains belonged to genotype I, subgenotypes Ia, Ib, and Ic, and were genetically close to Chinese viral strains reported in 2019–2023. Recombination events occurred in three of the Vietnamese DuCV strains, and one positive selection was found in the Rep/ORF3 gene.

## Figures and Tables

**Table 1:** General information on the sequences used in this study.

No.	GenBank Accession Number	Strain	Country	Host	Year	Genotype
1	OR842553.1	CN/GD/1092	China	Duck	2023	I
2	OR842554.1	CN/GD/1099	China	Duck	2023	I
3	PP860902.1	SDLC	China	Duck	2023	I
4	PP081932.1	Xinjiang2023	China	Duck	2023	I
5	OR134489.1	DuCV-GX51	China	Duck	2022	I
6	PP860900.1	SDHZ	China	Duck	2022	I
7	OQ657184.1	DZ1116	China	Duck	2022	I
8	OR134488.1	GX49	China	Duck	2022	I
9	OM176552.1	VNUA-HY40	Vietnam	Duck	2021	I
10	OM176553.1	VNUA-HN47	Vietnam	Duck	2021	I
11	OM176554.1	VNUA-TB61	Vietnam	Duck	2021	I
12	OM176555.1	VNUA-TN85	Vietnam	Duck	2021	I
13	ON227551.1	GD/JM/180	China	Duck	2021	I
14	ON227553.1	GD/ZQ/188	China	Duck	2021	I
15	ON227542.1	GD/QY/129	China	Duck	2021	I
16	ON227548.1	GD/ZQ/144	China	Duck	2021	I
17	OK094642.1	DY01	China	Duck	2020	I
18	OR134472.1	GX25	China	Duck	2020	I
19	OR134475.1	GX28	China	Duck	2020	I
20	OR134505.1	GX29	China	Duck	2020	I
21	OR134478.1	GX33	China	Duck	2020	I
22	OR134508.1	GX35	China	Duck	2021	I
23	OR134510.1	GX39	China	Duck	2021	I
24	OR134481.1	GX41	China	Duck	2021	I
25	OR134464.1	GX05	China	Duck	2019	I
26	OR134467.1	GX11	China	Duck	2019	I
27	MW255980.1	BaodingNO.Du	China	Duck	2019	I
28	MK814581.1	GX190510	China	Duck	2019	I
29	MT084134.1	SDHX1223	China	Duck	2018	I
30	MT084131.1	AHHF1109	China	Duck	2018	I
31	MN928795.1	HN02	China	Duck	2018	I
32	MN068356.1	HZ170301	China	Duck	2017	I
33	MN068357.1	YF180401	China	Duck	2017	I
34	MF627688.1	JSPX03E	China	Duck	2016	I
35	KY328304.1	SDFC12	China	Duck	2015	I
36	GU014543.1	AQ0901	China	Duck	2009	I
37	KC460531.1	NN12/2012	China	Duck	2012	I
38	HQ180265.1	LZ/11/09	China	Muscovy duck	2009	I
39	GU168779.1	FJzq290	China	Muscovy duck	2008	I
40	GQ334371.1	Zhejiang	China	Duck	2008	I
41	EU344803.1	LJ33	China	Duck	2007	I
42	EU344806.1	YS07	China	Muscovy duck	2007	I
43	MT318126.1	VC4	Brasil	Mallard	2014	I
44	KC851819.1	D12-KD-019	South Korea	Duck	2012	I
45	KC851821.1	D12-KD-028	South Korea	Duck	2012	I
46	DQ100076.1	33753-52	USA	Duck	2007	I
47	AY228555.1		Germany	Mallard duck	2003	I
48	NC_005053.1		Germany	Mallard duck	2003	I
49	OM176556.1	VNUA-HD89	Vietnam	Duck	2021	II
50	OM176557.1	VNUA-BG135	Vietnam	Duck	2021	II
51	MK814585.1	YN180506	China	Duck	2018	II
52	MN052853.1	FJ1815	China	Muscovy duck	2018	II
53	KR491945.1	YN24-2013	China	Muscovy duck	2013	II
54	KP229377.1	CP12021	Taiwan (China)	Muscovy duck	2012	II
55	GU131343.1	WF0804	China	Duck	2008	II
56	EU344805.1	MH11	China	Muscovy duck	2007	II
57	EU499309.1	MH02/07	China	Mule duck	2007	II
58	EU344802.1	HZ09	China	Muscovy duck	2007	II
59	DQ166836.1	TC2	Taiwan (China)	Muscovy duck	2002	II
60	DQ166838.1	TC4	Taiwan (China)	Muscovy duck	2002	II
61	OP432310.1	HNU-HYH	China	Duck	2022	III

**Table 2:** Diagnosis of duck circovirus in field samples using the PCR method.

Farm Number	Location	No. of Tested Samples	No. of Positive Samples	Positive Rate
1	Thainguyen	5	1	20
2	Bacgiang	5	2	40
3	Haiduong	5	2	40
4	Bacgiang	5	2	40
5	Nghean	5	1	20
6	Thainguyen	5	1	20
7	Phutho	5	3	60
8	Hanoi	5	3	60
9	Hanam	5	1	20
Total		45	16	35.56

**Table 3:** Nucleotide identities of the Vietnamese complete genomes in this study.

Virus Strain	Nucleotide Identity (%)								
	Vietnam/ VNUA-102/2023	Vietnam/ VNUA-114/2023	Vietnam/ VNUA-137/2023	Vietnam/ VNUA-225/2023	Vietnam/ VNUA-251/2023	Vietnam/ VNUA-318/2024	Vietnam/ VNUA-322/2024	Vietnam/ VNUA-315/2024	Vietnam/ VNUA-331/2024
Vietnam/VNUA-102/2023	100								
Vietnam/VNUA-114/2023	97.03	100							
Vietnam/VNUA-137/2023	97.03	99.19	100						
Vietnam/VNUA-225/2023	99.44	97.18	97.08	100					
Vietnam/VNUA-251/2023	96.93	99.19	99.49	97.08	100				
Vietnam/VNUA-318/2024	99.39	96.93	97.03	99.24	96.88	100			
Vietnam/VNUA-322/2024	97.13	99.29	99.19	97.29	99.29	97.08	100		
Vietnam/VNUA-315/2024	97.08	99.24	99.84	97.13	99.54	96.98	99.24	100	
Vietnam/VNUA-331/2024	97.29	99.34	99.44	97.34	99.44	97.19	99.34	99.49	100

**Table 4:** Comparison of nucleotide identities between current Vietnamese DuCV complete genomes and previous Vietnamese strains in GenBank.

Virus Strain	Nucleotide Identity (%)					
	Vietnam/OM176553.1/ VNUA-HN47/2021	Vietnam/OM176554.1/ VNUA-TB61/2021	Vietnam/OM176552.1/ VNUA-HY40/2021	Vietnam/OM176555.1/ VNUA-TN85/2021	Vietnam/OM176556.1/ VNUA-HD89/2021	Vietnam/OM176557.1/ VNUA-BG135/2021
Vietnam/VNUA-102/2023	96.98					
Vietnam/VNUA-114/2023	99.14	99.04				
Vietnam/VNUA-137/2023	99.54	99.44				
Vietnam/VNUA-225/2023	97.03	97.03	97.04			
Vietnam/VNUA-251/2023	99.34	99.24	97.54	96.98		
Vietnam/VNUA-318/2024	96.88	96.88	97.19	99.79	83.50	
Vietnam/VNUA-322/2024	99.14	99.04	97.64	97.19	83.70	84.44
Vietnam/VNUA-315/2024	99.59	99.49	97.49	97.08	83.40	84.04
Vietnam/VNUA-331/2024	99.39	99.29	97.79	97.29	83.65	84.19

**Table 5:** Nucleotide identity between the Vietnamese DuCV complete genomes and other sequences from abroad.

Strain Name	Virus with the Highest Nucleotide Identity				
	GenBank Accession Number	Strain Name	Country	Year	%
Vietnam/VNUA-102/2023	OK094642.1	DY01	China	2020	99.54
Vietnam/VNUA-114/2023	OR134472.1	GX25	China	2020	99.49
Vietnam/VNUA-137/2023	OR134489.1	GX51	China	2022	99.64
	OR842553.1	CN/GD/1092	China	2023	
Vietnam/VNUA-225/2023	OK094642.1	DY01	China	2020	99.24
Vietnam/VNUA-251/2023	OR134464.1	GX05	China	2019	99.59
	OR134467.1	GX11	China	2019	
Vietnam/VNUA-318/2024	OR134510.1	GX39	China	2021	99.64
	OK094642.1	DY01	China	2020	
Vietnam/VNUA-322/2024	OR134472.1	GX25	China	2020	99.49
Vietnam/VNUA-315/2024	OR134489.1	GX51	China	2022	99.69
Vietnam/VNUA-331/2024	OR134472.1	GX25	China	2020	99.74

**Table 6:** Amino acid substitutions on the cap protein of Vietnamese DuCV strains.

Virus Strain	Genotype	Amino Acid Residues on Cap Protein													
		12	47	55	82	106	107	155	156	177	183	194	197	205	236
Majority		G	N	S	Q	N	K	T	T	V	V	T	Y	R	D
**VNUA-102/2023**	I	.	H	.	R	S	T	.	A	.	I	G	H	K	N
**VNUA-114/2023**	I	.	.	.	.	.	.	.	.	.	.	.	.	.	.
**VNUA-137/2023**	I	.	.	N	.	.	.	S	.	.	.	.	.	.	.
**VNUA-225/2023**	I	.	H	S	R	S	T	.	A	.	I	G	H	K	N
**VNUA-251/2023**	I	.	.	N	.			S	.	.	.	.	.	.	.
**VNUA-318/2024**	I	.	H	.	R	S	T	.	.	.	I	G	H		N
**VNUA-322/2024**	I	.	.	.	.	.	.	.	.	I	.	.	.	.	.
**VNUA-315/2024**	I	.	.	N	.	.	.	S	.	.	.	.	.	.	.
**VNUA-331/2024**	I	.	.	.	.	.	.	.	.	.	.	.	.	.	.
VNUA-HN47/2021	I	.	.	N	.	.	.	.	.	.	.	.	.	.	.
VNUA-TB61/2021	I	S	.	N	.	.	.	.	.	.	.	.	.	.	.
VNUA-HY40/2021	I	.	.	.	.	S	T	.	.	.	.	.	.	.	.
VNUA-TN85/2021	I	.	H	.	R	S	T	.	.	.	I	G	H	K	N
VNUA-HD89/2021	II	A	H	.	.	.	.	.	.	I	.	G	.	K	E
VNUA-BG135/2021	II	A	H	.	.	G	.	.	.	I	.	G	.	K	E

Vietnamese DuCV strains in this study are indicated in bold.

**Table 7:** Recombination statistics of the Vietnamese DuCV Cap gene sequences using RDP 5.

Method	Recombination *p*-Value
GENECONV	3.71 × 10^−2^
MaxChi	3.26 × 10^−2^
Chimaera	3.16 × 10^−2^
SiScan	9.58 × 10^−5^
PhylPro	3.42 × 10^−2^

Recombination events having a *p*-value below 0.05 were deemed reliable.

**Table 8:** Recombination analysis of the Vietnamese DuCV Cap gene sequences using RDP 5.

No.	Recombination Event	Major Parent	Minor Parent
1	Vietnam/VNUA-102/2023	Vietnam/VNUA-114/2023	Germany/NC_005053.1/2003
2	Vietnam/VNUA-225/2023	Vietnam/OM176552.1/VNUA-HY40/2021	Germany/NC_005053.1/2003
3	Vietnam/VNUA-318/2024	Vietnam/OM176552.1/VNUA-HY40/2021	Germany/NC_005053.1/2003

**Table 9:** Positive selection on Rep protein of DuCV strains.

Site	α	β	β − α	Prob [α > β]	Prob [α < β]	Bayes Factor [α and β]
112	0.62	4.98	4.36	0.02	0.95	42.93

α indicates posterior synonymous substitution rate at a site; β indicates posterior non-synonymous substitution rate at a site; α > β: negative selection; α < β: positive selection; α = β: neutral selection; Prob [α > β] ≥ 0.9: posterior probability of negative selection at a site; Prob [α < β] ≥ 0.9: posterior probability of positive selection at a site. Eighty-nine negatively selective positions were indicated.

## Data Availability

The data presented in this study are available within the article. Raw data supporting this study are available from the corresponding author.

## Supplementary Materials

The following supporting information can be downloaded at https://www.mdpi.com/article/10.3390/v17050732/s1, Table S1: Comparison of nucleotide identity among Vietnamese DuCV strains in this study based on Cap gene sequences; Table S2: Comparison of nucleotide identity among Vitenamse DuCV strains in this study based on Rep gene sequences; Table S3: Negative selection on VP2 protein sequence.
