# Supplementary material for: Characterization of an Emerging Recombinant Duck Circovirus in Northern Vietnam, 2023–2024
Source: Viruses. 2025 May 20;17(5):732. doi: 10.3390/v17050732 (PMC12115742; doi:10.3390/v17050732)
Supplement: Supplementary file 1 [file viruses-17-00732-s001.zip › viruses-3611430-supplementary.pdf]

Table S1. Comparison of nucleotide identity among Vietnamese DuCV strains in this study based on Cap gene sequences

Strain name	VNUA-102/ 2023	VNUA-114/ 2023	VNUA-137/ 2023	VNUA-225/ 2023	VNUA-251/ 2023	VNUA-318/ 2024	VNUA-322/ 2024	VNUA-315/ 2024	VNUA-331/ 2024
VNUA-102/2023	100								
VNUA-114/2023	93.79	100							
VNUA-137/2023	93.28	98.70	100						
VNUA-225/2023	99.61	93.92	93.41	100					
VNUA-251/2023	93.41	98.83	99.61	93.54	100				
VNUA-318/2024	99.09	93.92	93.66	99.22	93.54	100			
VNUA-322/2024	93.79	98.70	98.44	93.92	98.57	93.92	100		
VNUA-315/2024	93.54	98.96	99.74	93.66	99.87	93.66	98.70	100	
VNUA-331/2024	93.92	99.35	99.09	94.05	99.22	94.05	99.09	99.35	100

Table S2. Comparison of nucleotide identity among Vietnamese DuCV strains in this study based on Rep gene sequences

Strain name	Vietnam/ VNUA-102/ 2023	Vietnam/ VNUA-114/ 2023	Vietnam/ VNUA-137/ 2023	Vietnam/ VNUA-225/ 2023	Vietnam/ VNUA-251/ 2023	Vietnam/ VNUA-318/ 2024	Vietnam/ VNUA-322/ 2024	Vietnam/ VNUA-315/ 2024	Vietnam/ VNUA-331/ 2024
Vietnam/VNUA-102/2023	100								
Vietnam/VNUA-114/2023	99.08	100							
Vietnam/VNUA-137/2023	99.54	99.31	100						
Vietnam/VNUA-225/2023	99.77	99.31	99.54	100					
Vietnam/VNUA-251/2023	99.20	99.20	99.20	99.43	100				
Vietnam/VNUA-318/2024	99.77	99.08	99.54	99.77	99.31	100			
Vietnam/VNUA-322/2024	99.31	99.54	99.54	99.54	99.65	99.43	100		
Vietnam/VNUA-315/2024	99.54	99.31	100.0	99.54	99.20	99.54	99.54	100	
Vietnam/VNUA-331/2024	99.54	99.08	99.54	99.54	99.43	99.54	99.31	99.54	100

Table S3. Negative selection on VP2 protein sequence

No.	Site	a	b	b-a	Prob [a>b]	Prob [a<b]	BayesFactor [a&b]
1	4	3.19	0.28	-2.90	0.95	0.04	0.08
2	8	9.82	0.28	-9.54	1.00	0.00	0.00
3	10	21.17	0.38	-20.79	1.00	0.00	0.00
4	13	2.46	0.28	-2.18	0.94	0.05	0.11
5	15	5.88	0.27	-5.61	1.00	0.00	0.01
6	17	6.89	0.31	-6.58	1.00	0.00	0.01
7	23	16.58	0.37	-16.22	1.00	0.00	0.00
8	24	24.00	0.38	-23.62	1.00	0.00	0.00
9	28	5.68	0.65	-5.03	0.98	0.01	0.02
10	30	2.90	0.23	-2.66	0.96	0.03	0.07
11	38	6.02	0.24	-5.78	1.00	0.00	0.01
12	39	3.26	0.27	-2.99	0.96	0.03	0.07
13	42	5.50	0.31	-5.19	0.99	0.01	0.02
14	44	12.86	0.35	-12.51	0.99	0.00	0.01
15	50	5.24	0.27	-4.96	0.98	0.02	0.03
16	58	5.27	0.30	-4.97	0.99	0.01	0.02
17	59	8.53	0.26	-8.27	1.00	0.00	0.00
18	61	2.90	0.28	-2.62	0.95	0.04	0.09
19	62	5.92	0.30	-5.62	0.99	0.01	0.01
20	64	4.38	0.33	-4.05	0.96	0.03	0.07
21	69	38.44	0.86	-37.58	1.00	0.00	0.00
22	85	5.22	0.30	-4.92	0.99	0.01	0.02
23	88	3.62	0.30	-3.32	0.95	0.03	0.08
24	99	2.34	0.25	-2.09	0.95	0.04	0.09
25	100	4.38	0.33	-4.05	0.96	0.03	0.07
26	103	16.75	0.37	-16.37	1.00	0.00	0.00
27	111	3.80	0.24	-3.57	0.99	0.01	0.02
28	113	7.34	0.62	-6.73	1.00	0.00	0.00
29	122	6.96	0.61	-6.35	0.99	0.01	0.01
30	123	3.88	0.30	-3.59	0.98	0.02	0.04
31	124	9.05	0.99	-8.05	0.98	0.01	0.01
32	129	7.48	0.29	-7.19	1.00	0.00	0.01
33	134	14.25	0.28	-13.97	1.00	0.00	0.00
34	144	2.21	0.27	-1.93	0.94	0.05	0.11
35	146	2.14	0.26	-1.88	0.94	0.05	0.11
36	147	3.87	0.23	-3.63	0.99	0.01	0.02
37	149	8.87	0.28	-8.59	1.00	0.00	0.00
38	152	8.82	0.63	-8.19	1.00	0.00	0.00
39	153	12.86	0.35	-12.51	0.99	0.00	0.01
40	154	5.24	0.27	-4.97	0.98	0.02	0.03
41	155	4.38	0.32	-4.06	0.92	0.06	0.15
42	160	11.36	0.62	-10.74	1.00	0.00	0.00
43	161	43.55	1.63	-41.92	1.00	0.00	0.00
44	162	14.99	0.33	-14.66	1.00	0.00	0.00

45	164	3.81	0.27	-3.54	0.98	0.01	0.03
46	165	5.47	0.29	-5.19	1.00	0.00	0.01
47	168	11.12	0.27	-10.85	1.00	0.00	0.00
48	169	14.79	0.28	-14.51	1.00	0.00	0.00
49	170	5.85	0.69	-5.16	0.96	0.02	0.05
50	174	3.35	0.28	-3.07	0.95	0.04	0.08
51	177	10.57	0.30	-10.27	1.00	0.00	0.00
52	179	4.37	0.32	-4.05	0.92	0.06	0.15
53	207	2.34	0.31	-2.04	0.90	0.08	0.18
54	213	9.70	0.26	-9.44	1.00	0.00	0.00
55	214	5.80	0.63	-5.17	0.96	0.02	0.05
56	216	4.86	0.68	-4.19	0.92	0.05	0.12
57	217	2.73	0.28	-2.45	0.95	0.04	0.10
58	218	2.08	0.27	-1.81	0.93	0.05	0.12
59	219	13.56	0.34	-13.22	1.00	0.00	0.00
60	222	3.14	0.30	-2.84	0.95	0.04	0.09
61	224	5.97	0.63	-5.33	0.96	0.02	0.05
62	225	11.73	0.27	-11.46	1.00	0.00	0.00
63	226	9.23	0.32	-8.92	1.00	0.00	0.00
64	227	6.21	0.27	-5.94	0.99	0.00	0.01
65	229	4.89	0.32	-4.57	0.93	0.06	0.13
66	230	16.20	0.29	-15.91	1.00	0.00	0.00
67	235	2.21	0.27	-1.94	0.94	0.05	0.11
68	240	6.55	0.32	-6.23	0.94	0.04	0.10
69	241	10.32	0.30	-10.03	1.00	0.00	0.00
70	246	7.86	0.30	-7.56	1.00	0.00	0.00
71	249	25.13	0.41	-24.72	1.00	0.00	0.00
72	254	10.50	0.32	-10.19	1.00	0.00	0.00
73	259	3.15	0.30	-2.85	0.95	0.04	0.09
74	260	23.47	0.95	-22.52	1.00	0.00	0.00
75	261	4.19	0.26	-3.93	0.97	0.02	0.05
76	262	2.25	0.27	-1.98	0.94	0.05	0.11
77	263	2.14	0.29	-1.85	0.93	0.06	0.13
78	264	3.15	0.28	-2.86	0.95	0.04	0.09
79	266	8.33	0.32	-8.02	0.99	0.01	0.02
80	269	6.59	0.32	-6.28	0.94	0.04	0.10
81	270	3.27	0.28	-2.99	0.95	0.04	0.08
82	272	10.88	0.31	-10.56	1.00	0.00	0.00
83	274	3.14	0.30	-2.85	0.95	0.04	0.09
84	276	3.46	0.30	-3.16	0.95	0.04	0.09
85	280	4.40	0.54	-3.86	0.96	0.03	0.07
86	287	1.86	0.24	-1.62	0.90	0.07	0.18
87	288	3.14	0.24	-2.91	0.96	0.03	0.06
